# Efficacy of Surgical/Wound Washes against Bacteria: Effect of Different In Vitro Models

**DOI:** 10.3390/ma15103630

**Published:** 2022-05-19

**Authors:** Farhana Parvin, Karen Vickery, Anand K. Deva, Honghua Hu

**Affiliations:** Faculty of Medicine, Health and Human Sciences, Macquarie University, Sydney 2109, Australia; mst-farhana.parvin@hdr.mq.edu.au (F.P.); karen.vickery@mq.edu.au (K.V.); anand.deva@mq.edu.au (A.K.D.)

**Keywords:** biofilm, antiseptics, surgical washes, wound washes, in vitro models, infection

## Abstract

Topical antiseptics are often used to treat chronic wounds with biofilm infections and during salvage of biofilm contaminated implants, but their antibacterial efficacy is frequently only tested against non-aggregated planktonic or free-swimming organisms. This study evaluated the antibacterial and antibiofilm efficacy of four commercial surgical washes Bactisure, TorrenTX, minimally invasive lavage (MIS), and Betadine against six bacterial species: *Staphylococcus epidermidis*, *Staphylococcus aureus*, *Streptococcus pyogenes*, *Acinetobacter baumannii*, *Pseudomonas aeruginosa,* and *Escherichia coli,* which are commonly isolated from surgical site infections and chronic wound infections using different in vitro models. We determined minimum planktonic inhibitory and eradication concentration and minimum 1-day-old biofilm inhibition and eradication concentration of antiseptics in 96-well plates format with 24 h contact time. We also tested the efficacy of antiseptics at in-use concentration and contact time in the presence of biological soil against 3-day-old biofilm grown on coupons with shear in a bioreactor, such that the results are more applicable to the clinical biofilm situations. In the 96-well plate model, the minimum concentration required to inhibit or kill planktonic and biofilm bacteria was lower for Bactisure and TorrenTX than for MIS and Betadine. However, Betadine and Bactisure showed better antibiofilm efficacy than TorrenTX and MIS in the 3-day-old biofilm bioreactor model at in-use concentration. The minimal concentration of surgical washes required to inhibit or kill planktonic bacterial cells and biofilms varies, suggesting the need for the development and use of biofilm-based assays to assess antimicrobial therapies, such as topical antiseptics and their effective concentrations. The antibiofilm efficacy of surgical washes against different bacterial species also varies, highlighting the importance of testing against various bacterial species to achieve a thorough understanding of their efficacy.

## 1. Introduction

Surgical washes/antiseptics have been used to prevent contamination of implants and to treat wound infection, particularly superficial wounds of the extremities. Wounds, especially chronic wounds, affect millions of patients worldwide. Chronic wounds include pressure ulcers, venous stasis ulcers, diabetic foot ulcers, decubitus ulcers, infected medical devices, and non-healing surgical wounds. They pose a significant burden on healthcare facilities, increase the cost of care, increase morbidity and mortality, resulting in decreased patient quality of life and productivity. It is estimated that around 8.2 million people in the USA have chronic wounds, which costs between USD 28.1 to USD 96.8 billion per annum in direct medical costs [1].

Implants and wounds become contaminated with organisms from the surrounding skin, other endogenous patient sources, and the local environment resulting in colonization by diverse microbes, many of which are potentially pathogenic [2,3]. Identification of biofilm bacteria using molecular approaches shows that hundreds of bacterial species can contaminate chronic wounds [4]. Once colonized, a wound can become infected, and the resulting tissue damage and release of bacterial toxins and enzymes lead to the recruitment of inflammatory cells and delayed healing or non-healing [5,6,7]. 

Topically applied surgical washes and antiseptics and wound dressings are regularly applied for wound management. Antiseptics are biocides that either destroy or inhibit the growth of microorganisms in or on living tissue [8] and usually have a microbicidal effect and broader antimicrobial activity than antibiotics [9], but they must balance antibacterial activity with the tolerance constraints of living tissue. A perfect wound cleanser would only cause minimum harm to healthy tissues yet eliminate microorganisms, cause no sensitization, stay effective in the presence of organic material, as well as being stable and inexpensive [10,11]. The actions of antiseptics include protein coagulation and precipitation, cell wall or membrane permeability changes, and specific or generalized toxicity to bacterial enzymatic systems [12].

Unfortunately, the microenvironment of chronic wounds provides an ideal habitat for bacteria to form biofilm [13]. Collagen, fibronectin, and laminin are extracellular matrix components that provide attachment for bacteria [14] while the wound bed environment is hydrated, nutrient-rich, and at a suitable pH for bacterial growth. Biofilms form in up to 90% of chronic wounds and are thought to be a critical player leading to wound chronicity [15]. Wound biofilms are an aggregate of microbes enclosed in a self-produced matrix of extracellular polymeric substance (EPS) and can be found superficially and deep in the wound bed [16]. To improve wound healing, removing bacterial biofilm is a priority, and investigation of antibiofilm agents that can efficiently minimize and eradicate biofilm-related infections is desperately needed.

Biofilm bacteria are more tolerant to antimicrobial agents than planktonic bacteria [17,18] and older biofilms are generally more resistant and harder to kill than less mature biofilms [18,19,20]. Nevertheless, surgical washes/antiseptics are generally evaluated against planktonic cells to determine their efficacy [21]. The standard method for testing antibiotic therapy is the determination of the minimum inhibitory concentration (MIC) and the minimum eradication concentration (MEC) and involves testing various antibiotic concentrations against a set number of bacteria for 24 h [22]. The basis for this contact time is that antibiotic therapy is given continuously, usually over days, but 24 h is sufficient time to determine if the bacteria is resistant to the test compound. However, antiseptics need to penetrate the biofilm EPS to kill the enclosed bacteria within the much shorter contact time recommended and used in clinical situations.

In vitro efficacy testing of antiseptics against biofilms is most frequently determined using the MBEC (Calgary device) or microtiter plate format [23] and often the tested contact time is 24 h. However, we have previously shown that antiseptic efficacy can vary with both the presence or absence of soil, the bacterial species tested, and the type of in vitro model used [24]. 

We hypothesize that in chronically infected wounds and contaminated implants, the combined EPS and patient secretions are likely to prevent antiseptics from working optimally; therefore, surgical washes need to be able to quickly penetrate this combined soil and kill the bacteria within a short contact time in clinical situations.

This study aimed to evaluate the antibacterial and antibiofilm efficacy of four different wound washes on six bacterial species commonly isolated from surgical site infections and chronic wound infections using different in vitro models.

## 2. Materials and Methods

### 2.1. Bacterial Strains

The efficacy of antimicrobial washes was tested against three Gram-positive species: *Staphylococcus epidermidis* (ATCC 35984), *Staphylococcus aureus* (ATCC 25923), and *Streptococcus pyogenes* (ATCC 8668), and three Gram-negative species: *Pseudomonas aeruginosa* (ATCC 25619), *Escherichia coli* K12 (ATCC 23724), and *Acinetobacter baumannii* (ATCC 19606).

### 2.2. Antimicrobial Washes

The composition of Bactisure, TorrenTX, and minimally invasive lavage (MIS) manufactured by Next Science IP Holding Pty Ltd., Sydney, Australia, is shown in Table 1. These antimicrobials were compared with Betadine solution, 10% *w*/*v* povidone iodine (Livingstone, Sydney, Australia), which is used routinely in clinics for surgical irrigation and wound cleaning.

### 2.3. Determining Minimum Planktonic Inhibitory and Eradication Concentration, Minimum Biofilm Inhibition, and Eradication Concentration

The minimum inhibitory concentration (MIC), minimum eradication concentration (MEC), minimum biofilm inhibition (MBIC), and minimum biofilm eradication concentration (MBEC) was determined by 2-fold dilution of the antiseptic to inhibit or eradicate the 10^7^ planktonic bacteria cells or biofilm in 96-well plates using our published method [24].

A colony of each bacterium grown in 100% tryptone soya broth (TSB, Thermo Fisher Scientific, Waltham, MA, USA) at 37 °C with 130 rpm for 16 h (overnight) resulted in early stationary phase culture with around 10^9^/mL bacterial cells. This culture was diluted in TSB to give 0.3 absorbance at 600 nm (~10^8^ cells/mL).

For MIC determination, an aliquot of 100 µL of 10^8^ bacterial cells/mL (10^7^ cells) was added to each well in 11 columns of a 96-well plate (except column 2 was added TSB only as negative control). An amount of 100 µL of 2-fold serially diluted antiseptics was added to each well (8 wells/column/dilution) or 100 µL of water was added to column 1 (positive control) and column 2 (negative control) of 96-well plates and incubated at 37 °C for 24 h. The MIC was the lowest concentration of the test agent that prevents bacterial growth as determined by the lack of turbidity in all 8 wells in the corresponding column. For MEC: 20 µL of each well from MIC plate was transferred to a fresh plate containing 180 µL of TSB (growth media) and incubated at 37 °C for another 24 h. The MEC was the lowest concentration of the test agent that eradicated bacteria (no recovery growth by lack of turbidity in all 8 wells in the corresponding column). Turbidity was assessed visually and at 600 nm wavelength in a microplate reader (PHERAstar FS, BMG Labtech, Ortenberg, Germany).

For MBIC determination, biofilm was formed by adding 100 µL of 10^6^ bacterial cells/mL (10^5^ cells) to each well (except in column 2, TSB was added only as the negative control) of a microtiter plate and incubated for 24 h at 37 °C which gave approximately 10^7^ biofilm cells attached to the well sides as determined by CFU count. The media was removed and 100 µL TSB and 100 µL of diluted antiseptic was added as detailed for the MIC determination above prior to 24 h incubation. The MBIC was the lowest concentration of antiseptic required to inhibit biofilm growth and the release of planktonic organisms into the media was determined by lack of turbidity. For each well of the MBIC plate that showed no growth, a 20 µL aliquot was transferred to 180 µL fresh TSB in a clean 96-well plate and incubated for another 24 h. The MBEC was the lowest concentration of test agent that eradicated bacteria (no recovery growth by lack of turbidity in all 8 wells in the corresponding column).

As the antimicrobial washes were added to an equal volume of bacterial culture, the concentration tested was half the concentration of antiseptic added to each well; therefore, the maximum concentration of product in these tests was half the recommended in-use concentration as the products were provided ready-for-use. Each antiseptic was tested in triplicates in two independent experiments. If the independent experiments results were different, then the higher concentration was reported in the results.

### 2.4. Efficacy against 3-Day-Old Mature Biofilm at In-Use Concentration and Contact Time in the Presence of Biological Soil

Biofilm was grown on 24 polycarbonate coupons in the Centers for Disease Control (CDC) biofilm reactor (BioSurface Technologies Corp., Bozeman, MT, USA). The reactor was inoculated with 10^8^ bacteria/mL in 500 mL of 50% TSB under shear (130 rpm) at 35 °C in batch phase for 48 h, at which time the media was removed and replaced with 20% TSB and incubated for a further 24 h according to [24]. Each polycarbonate coupon was covered with approximately 10^7^ bacterial cells as determined by CFU count. Coupons were washed in 10 mL phosphate-buffered saline (PBS) to remove loosely attached planktonic bacteria. Biofilm covered coupons (*n* = 6/test group) were immersed in 2 mL of test products for the manufacturer’s specified concentration and contact time (Bactisure: 1 min and 3 min; Betadine solution: 5 min; TorrenTX: 10 min; minimally invasive lavage (MIS): 60 min) for each antiseptic in the presence of soil provided by 5% bovine calf serum (BCS, Sigma-Aldrich). Antimicrobial action was halted by coupons being washed in 5 mL PBS twice followed by socking in Dey–Engley (D/E) neutralizer broth (Thermo Fisher Scientific, Waltham, MA, USA), for 10 min. The coupons were then individually placed in 2 mL of PBS and sonicated in an ultrasonic bath (Soniclean; Dudley Park, SA, Australia) for 10 min at 42–47 kHz followed by a 2 min vortex prior to standard plate culture and colony forming units (CFU) count. 

CFU log_10_ reduction was calculated as the CFU Log_10_ value of control (without treatment) for each bacterial species minus the CFU Log_10_ value of each testing antiseptic. Each antiseptic was tested in triplicates in two independent experiments. The standard deviation was calculated from CFU Log_10_ of the six replicates in each antiseptic test.

### 2.5. Statistical Analysis

Statistical analysis was conducted using the statistical package Sigma Plot 13 (Systat Software, Inc., San Jose, CA, USA). The Shapiro–Wilk test was used to check if data were normally distributed. If normally distributed, the one-way analysis of variance (ANOVA) was used, or a Kruskal–Wallis ANOVA on ranks was used if the data was not normally distributed, and Tukey’s pairwise multiple comparisons tests were used to compare the viable bacteria number between different treatment groups against 3-day-old biofilms. *p* value < 0.05 was considered statistically significant.

## 3. Results

### 3.1. Minimum Planktonic Inhibitory and Eradication Concentration and Minimum Biofilm Inhibition and Eradication Concentration

**Figure 1:**
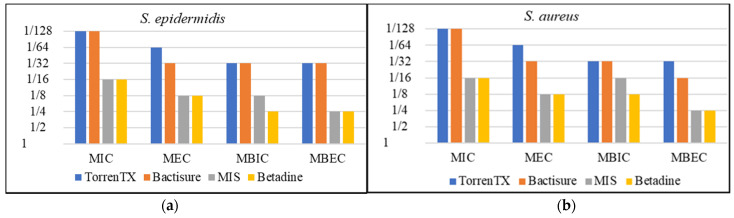

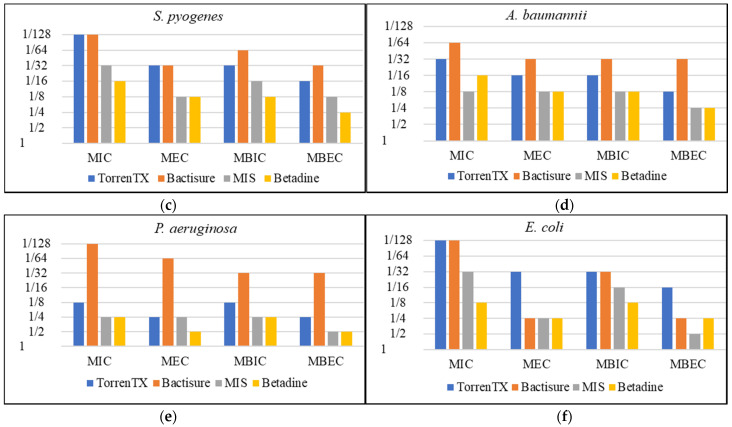
Effect of antimicrobial wound washes on inhibiting growth (MIC) and killing (MEC) planktonic microorganisms and inhibiting growth (MBIC) and killing (MBEC) 1-day-old bacterial biofilms of *S. epidermidis* (**a**), *S. aureus* (**b**), *S. pyogenes* (**c**), *A. baumannii* (**d**), *P. aeruginosa* (**e**) and *E. coli* (**f**).

Minimum planktonic inhibitory (MIC) and eradication concentration (MEC) and minimum biofilm inhibition (MBIC) and eradication concentration (MBEC) are summarized in Table 2 and graphically displayed in Figure 1.

MIC, MEC, MBIC, and MBEC tests based on 24 h contact time and serial dilution of antiseptics in comparison with Betadine solution (10% *w*/*v* povidone iodine) showed that lower or the same concentration of Bactisure and TorrenTX was required to inhibit or kill planktonic bacterial cells or biofilm against all six bacterial species tested than Betadine. The minimal concentration of MIS required to inhibit or kill planktonic bacterial cells or biofilm against bacterial species tested was the same or lower than Betadine in most cases, except in the case of MIC against *A. baumannii* and MBEC against *E. coli*, in which 2-fold greater concentration of MIS than Betadine was required.

The results showed that higher or the same concentration was required to inhibit or eradicate biofilm than to inhibit or eradicate planktonic cells for all the antiseptics tested.

However, MIC, MEC, MBIC, and MBEC do not represent the clinical in-use situation in terms of contact time and concentration. Therefore, we explored these antiseptics further against 3-day-old biofilms of each bacterial species in the presence of biological soil at in-use concentration and clinically based contact time.

### 3.2. Efficacy against 3-Day-Old Mature Biofilm at In-Use Concentration and Contact Time in the Presence of Biological Soil

**Figure 2:**
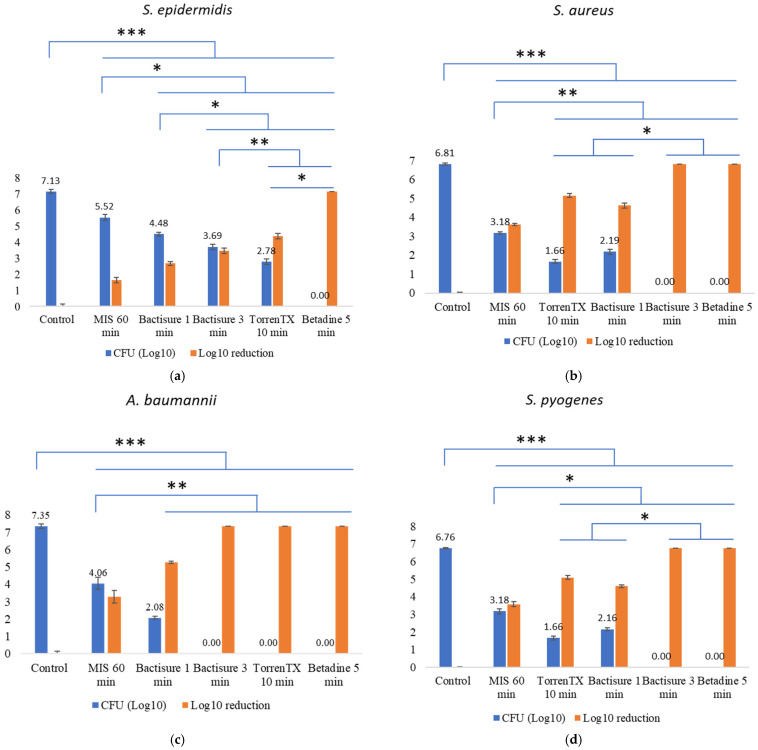

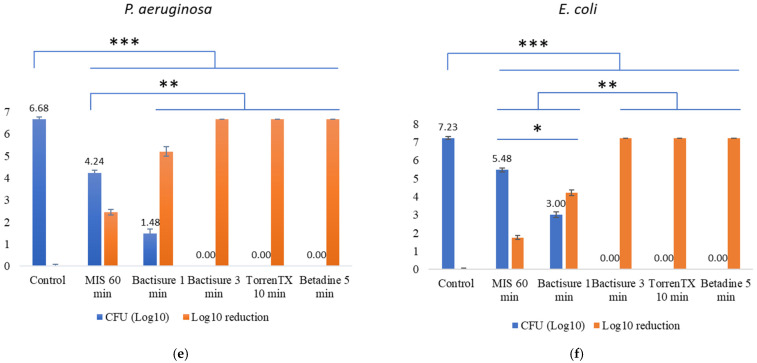
CFU (Log_10_) and CFU log_10_ reduction in 3-day-old bacterial biofilm cells for *S. epidermidis* (**a**), *S. aureus* (**b**), *A. baumannii* (**c**), *S. pyogenes* (**d**), *P. aeruginosa* (**e**), and *E. coli* (**f**) following treatment with Betadine, Bactisure, TorrenTX, and MIS at in-use concentration and contact time in the presence of 5% BCS as biological soil. Error bar represents standard deviation from CFU Log_10_ of the six replicates in each antiseptic test. * *p* < 0.05, ** *p* < 0.01, *** *p* < 0.001.

The efficacy of antiseptics tested against 3-day-old biofilms at in-use concentration and clinically based contact time in the presence of 5% BCS as biological soil is displayed in Figure 2. With a short contact time of 5 min, 10% *w*/*v* Betadine solution killed all biofilm bacteria of all six bacterial species tested. Bactisure treatment for 3 min eliminated all biofilm bacteria of five bacterial species except *S. epidermidis* biofilm. 

Although TorrenTX with a 10 min contact time effectively killed all the Gram-negative biofilm bacteria—*E. coli*, *P. aeruginosa*, and *A. baumannii*—it could not completely kill all the Gram-positive biofilm cells, with *S. epidermidis* being the most resilient. TorrenTX reduced the titre of *S. aureus* and *S. pyogenes* over 4 Log_10_.

MIS resulted in 3 Log_10_ reduction in *A. baumannii, S. aureus,* and *S. pyogenes,* but only resulted in over 2 Log_10_ reduction *in P. aeruginosa* and less than 2 Log_10_ reduction in *S. epidermidis* and *E. coli*.

## 4. Discussion

The efficacy of surgical washes is usually assessed by determining the MIC and MEC. Antimicrobial concentrations that kill rapidly growing planktonic cells frequently fail against biofilm infections. Many studies have focused on testing antibiofilm products, but up until recently, standardised biofilm methods have existed only for *P. aeruginosa* [25] and *S. aureus* [26]. The lack of standardisation and use of biofilm models not representing real-life situations, particularly in the wound bed [27,28,29], has hampered physicians’ decision making, especially considering that randomised clinical trials comparing different surgical washes are few. A recent review of the literature found that only three clinical studies, comparing surgical wash efficacy in chronic wounds, used appropriate techniques for confirming the presence of biofilm in the wound. The same study found that in vitro testing of antiseptics registered for human use was most frequently by MBEC determination using the Calgary device/microplate followed by the CDC biofilm reactor and log reduction calculations [23]. 

A variety of microbial strains including *S. aureus*, *S. epidermidis*, *P. aeruginosa*, *A. baumannii*, *E. coli*, and *S. pyogenes* were evaluated because of their relevance in implant and wound infections [3,30,31,32]. By testing the performance of these surgical washes on planktonic and in different biofilm models, we revealed that various bacteria respond to, and are affected by, antimicrobial solutions very differently. Comparing wound wash efficacy in these in vitro settings reveals which product works best against a specific bacterial species or biofilm.

From our MIC, MEC, MBIC, and MBEC study results, it is apparent that nearly all the tested antiseptics inhibit and eradicate all the six bacterial strains either in planktonic form or in biofilm state with 24 h contact time. In general, both planktonic and biofilm bacteria were inhibited at a lower concentration than that required for eradication (100% kill). The concentration of antiseptic to kill 1-day-old biofilm bacteria was greater than (in most cases) or the same as the concentration to kill planktonic bacteria or to inhibit biofilm growth.

In the 96-well plate model, the minimum concentration required to inhibit or kill planktonic and biofilm bacteria was lower for Bactisure and TorrenTX than for MIS and Betadine. Both Bactisure and TorrenTX have benzalkonium chloride acting as a surfactant and antimicrobial agent and ethanol 10% *w*/*v* acting principally as a solvent phase polarity modifier, but it may also have an antimicrobial effect under prolonged incubation as occurred during MIC, MEC, MBIC, and MBEC determinations. Benzalkonium chloride is one of the commonly used antiseptics reported in earlier studies [33,34]. The difference is that Bactisure has acetic acid and TorrenTX has citric acid as pH modifiers and chelating agents. 

Chelating agents play a crucial role in destabilising the biofilm structure and additionally impairing the stability of the bacterial membrane [35]. Chelators such as EDTA, citrate, etc., have been shown to prevent biofilm development by chelating metallic cations, which are required for bacterial cell proliferation and adhesion of microorganisms to fibrin and protein [36,37]. Both acetic acid and citric acid have been reported to have antibacterial and antibiofilm efficacy against different microorganisms [38,39,40]. The minimum concentration of Bactisure (containing acetic acid) and TorrenTX (containing citric acid) required to inhibit and eradicate Gram-positive bacteria was the same or only with a 2-fold difference, but up to 16-fold difference against the Gram-negative bacteria. Acetic acid has previously been shown to kill *P. aeruginosa* and *S. aureus* biofilms in both flow-through and a microplate assay at 0.5% and 1%, respectively [38]. Double the concentration of Bactisure (containing acetic acid) was required to kill *S. aureus* biofilm compared with *P. aeruginosa* (1/16 dilution compared with a 1/32 dilution). A 1/32 dilution of Bactisure contains only 0.175% of acetic acid, while a 1/4 dilution of TorrenTX, necessary to kill *P. aeruginosa,* contains 2.047% citric acid. Given that Bactisure and TorrenTX have the same active ingredients, these results confirm how important the formulation is to biocide efficacy when targeting biofilm. This has been demonstrated previously by Chowdhury, who showed that the addition of surfactants and chelating agents to hydrogen peroxide/peracetic acid disinfectant (Surfex) increased biofilm killing over 1000-fold compared with hydrogen peroxide/peracetic acid alone [41]. However, the effect of formulation on efficacy may vary from biofilm to biofilm, as TorrenTX containing citric acid was much more efficient at killing *E. coli.* This suggests efficacy testing should be conducted against multiple species of bacteria, not just one or two species.

Minimally invasive lavage contains sodium lauryl sulphate, which is a surfactant and antimicrobial. Sodium lauryl sulphate has previously been shown to have antimicrobial effects [42]. Around 78% *S. aureus* biofilm bacteria was removed using 0.5 mM sodium lauryl sulphate [42]. Minimally invasive lavage uses citric acid and sodium citrate as pH modifiers and metal chelators, but both are about 40% of the concentration as in TorrenTX. Sodium citrate is one of the components of minimally invasive lavage, which has been found to inhibit the biofilm formation of various *Staphylococcus* species in vitro [37]. The lower concentration of citric acid in minimally invasive lavage might be one of the reasons for its lower efficacy. Previous study showed an increased concentration of citric acid is needed for the removal of 24 h biofilm [40]. 

Over the 24 h contact time, Betadine, which is 10% povidone iodine, required a stronger concentration than either Bactisure or TorrenTX to inhibit or kill planktonic and 1-day-old biofilm. This is consistent with previous findings that the antimicrobial activity of benzalkonium chloride-based antiseptics was higher than povidone iodine when the contact time was 24 to 48 h [43]. In contrast, when used at the recommended in-use concentration, povidone iodine has been shown to be more efficient at eradicating bacterial biofilm than its counterparts [44]. The use of povidone iodine over long contact periods is controversial as an in vitro study found povidone iodine adversely affects wound healing, but this effect is reduced at lower povidone iodine concentrations [45].

From MIC, MEC, MBIC, and MBEC results, Bactisure containing acetic acid was more effective against *P. aeruginosa* than TorrenTX without acetic acid despite TorrenTX having the same or better efficacy against the other bacterial species tested. The sensitivity of *P. aeruginosa* to acetic acid has previously been reported [46,47,48]. Recent study showed that weak acid N-acetyl-L-cysteine and acetic acid can penetrate the *P. aeruginosa* biofilm matrix and kill 100% of bacteria cells embedded inside biofilm at pH < 3.5 [49]. 

The management of chronic wounds encompasses a wide range of factors. Although there is sufficient evidence that the efficacy of topical antimicrobial agents has in vitro effects on planktonic and biofilm microorganisms, MIC, MEC, MBIC, and MBEC do not represent the clinical in-use situation in terms of contact time and concentration. In vitro models studying topical antimicrobial wound treatments have not considered the clinical usage pattern of these products or the instructions in terms of duration of exposure, and results are generally published after 24 h exposure periods [50]. Furthermore, the use of immature biofilms (formed within 24 h) with relatively irregular structures, active metabolism, and less obvious stress response cannot represent the truly complex, mature, and highly resistant biofilms that have been seen in many wound infections [32,51]. Therefore, we measured the efficacy of the surgical washes against 3-day-old biofilms of each bacterial species in the presence of biological soil with an in-use concentration and realistic clinically based contact time.

Our findings showed that Betadine (povidone iodine, 10%) with 5 min exposure time was able to completely eradicate the 3-day-old biofilm bacteria of all six bacterial species tested in the presence of 5% bovine calf serum. Previous findings have also reported that Betadine was effective against mature biofilm with short exposure times [24,49]. Betadine has rapid, potent, broad-spectrum antimicrobial properties being active against both Gram-negative and Gram-positive bacteria, bacterial spores, protozoa, fungi, and various viruses [52]. It delivers free iodine into the cell, which interrupts the cellular process and results in bacterial death [53]. Additionally, it destroys the structural elements of the cell membrane [52].

Both Bactisure and TorrenTX also eradicated (100% kill) 3-day-old biofilm of all three Gram-negative bacteria biofilms tested *A. baumannii*, *P. aeruginosa,* and *E. coli*. The efficacy of TorrenTX and Bactisure varied although both products contain benzalkonium chloride as the main ingredient. Bactisure took just 3 min to kill all biofilm bacteria of five bacterial species tested except *S. epidermidis*. In contrast, TorrenTX with 10 min contact time only eradicated the Gram-negative organisms *P. aeruginosa, A. baumannii, and E. coli,* but not Gram-positive organisms *S. aureus*, *S. epidermidis,* and *S. pyogenes.* Overall, Bactisure proved to be superior to that of TorrenTX. 

Although MIS did not perform as well as the other products against 3-day-old biofilms, it was still capable of eradicating 1-day-old biofilm of all six bacterial species tested at 1/2 in-use concentration with 24 h contact time. As MIS causes no harm to the human body, therefore, it could be left in surgical pockets to continue its antibacterial and antibiofilm activity. Evaluating the antiseptic in vivo [54] would be the next step in a future study.

Overall, Bactisure and TorrenTX exhibited significant antibacterial and antibiofilm efficacy in MIC, MEC, MBIC, and MBEC tests with 24 h contact time. An increased contact time would be possible if these products were included in a wound dressing.

In conclusion, the current research emphasised the importance of additives such as chelating agents on antiseptic efficacy and how the efficiency of the antiseptics varied depending on the biofilm model used and the growth conditions of the biofilm, highlighting the importance of using a variety of biofilm model systems against multiple bacterial species when evaluating the efficacy of surgical/wound washes.

## Figures and Tables

**Table 1:** Composition of antiseptic formulations.

Product	Ingredients	g/L	Function
Bactisure	Benzalkonium chloride	1.30	Surfactant/antimicrobial
	Sodium acetate trihydrate	30.20	pH modifier—metal chelator (biofilm dissolution)
	Glacial acetic acid	59.00	pH modifier—metal chelator (biofilm dissolution)
	Ethanol	100.00	Solvent phase polarity modifier
	Water	807.00	Vehicle
TorrenTX wound wash	Benzalkonium chloride	1.30	Surfactant/antimicrobial
	Sodium citrate dihydrate	85.00	pH modifier—metal chelator (biofilm dissolution)
	Citric acid monohydrate	81.70	pH modifier—metal chelator (biofilm dissolution)
	Ethanol	100.00	Solvent phase polarity modifier
	Water	795.00	Vehicle
Minimally invasive lavage	Sodium lauryl sulphate	1.00	Surfactant/antimicrobial
	Sodium citrate dihydrate	35.70	pH modifier—metal chelator (biofilm dissolution)
	Citric acid anhydrous	32.50	pH modifier—metal chelator (biofilm dissolution)
	Water	963.80	Vehicle

**Table 2:** Maximum possible dilution of surgical washes to give MIC, MEC, MBIC, and MBEC based on 24 h contact time.

Antimicrobials	Bacterial Strains	MIC	MEC	MBIC	MBEC
Bactisure	*S. aureus*	1/128	1/32	1/32	1/16
	*S. epidermidis*	1/128	1/32	1/32	1/32
	*S. pyogenes*	1/128	1/32	1/64	1/32
	*P. aeruginosa*	1/128	1/64	1/32	1/32
	*A. baumannii*	1/64	1/32	1/32	1/32
	*E. coli*	1/128	1/4	1/32	1/4
TorrenTX wound wash	*S. aureus*	1/128	1/64	1/32	1/32
	*S. epidermidis*	1/128	1/64	1/32	1/32
	*S. pyogenes*	1/128	1/32	1/32	1/16
	*P. aeruginosa*	1/8	1/4	1/8	1/4
	*A. baumannii*	1/32	1/16	1/16	1/8
	*E. coli*	1/128	1/32	1/32	1/16
Minimally invasive lavage	*S. aureus*	1/16	1/8	1/16	1/4
	*S. epidermidis*	1/16	1/8	1/8	1/4
	*S. pyogenes*	1/32	1/8	1/16	1/8
	*P. aeruginosa*	1/4	1/4	1/4	1/2
	*A. baumannii*	1/8	1/8	1/8	1/4
	*E. coli*	1/32	1/4	1/16	1/2
Betadine solution	*S. aureus*	1/16	1/8	1/8	1/4
	*S. epidermidis*	1/16	1/8	1/4	1/4
	*S. pyogenes*	1/16	1/8	1/8	1/4
	*P. aeruginosa*	1/4	1/2	1/4	1/2
	*A. baumannii*	1/16	1/8	1/8	1/4
	*E. coli*	1/8	1/4	1/8	1/4

## Data Availability

The data generated are presented in this paper.
